# Harmonization of quantitative liver function evaluation using gadoxetate disodium-enhanced magnetic resonance imaging

**DOI:** 10.1007/s00330-025-11582-5

**Published:** 2025-04-18

**Authors:** Eriko Yoshizawa, Akira Yamada, Yukinori Okajima, Tsuyoshi Notake, Akira Shimizu, Yuji Soejima, Yasunari Fujinaga

**Affiliations:** 1 Department of Radiology, Shinshu University School of Medicine, Matsumoto, Japan; 2 Medical Data Science Course, Shinshu University School of Medicine, Matsumoto, Japan; 3 Division of Gastroenterological, Hepato-Biliary-Pancreatic, Transplantation and Pediatric Surgery, Department of Surgery, Shinshu University School of Medicine, Matsumoto, Japan

**Keywords:** Magnetic resonance imaging, Contrast media, Liver function test, Indocyanine green, Gadoxetic acid disodium

## Abstract

**Objectives:**

This study aimed to develop a clinically applicable harmonization method for the hepatocellular uptake index (HUI), a quantitative liver function index, using gadoxetate disodium-enhanced (EOB)-MRI, to ensure consistency across diverse MR systems.

**Materials and methods:**

This retrospective study, approved by our institutional review board, included consecutive patients who underwent three-dimensional gradient-echo T1-weighted EOB-MRI, HUI measurements, indocyanine green disappearance rate (ICG-PDR), and albumin-bilirubin linear predictor (ALBI-LP) between April 2011 and June 2024. Six different MR systems were used for HUI measurements. A harmonization method using ALBI-LP was developed and validated for estimating liver reserves corresponding to ICG-PDR through statistical analysis of residuals.

**Results:**

A total of 498 patients (mean age, 68.0 years ± 11.6; 320 men) were evaluated. A statistically significant linear correlation was observed between HUI, ICG-PDR, and ALBI-LP in each MR system, leading to the determination of conversion factors for HUI harmonization. The harmonizing equation, harmonized HUI (h-HUI) = HUI･(Slope2’/−1.425)･0.955, was derived, with Slope2’ representing the regression slope between HUI and ALBI-LP for each MR system. The standard deviation of the estimation error for ICG-PDR was significantly smaller using h-HUI by ALBI-LP (0.051, [0.048–0.054]) compared to non-harmonized HUI (0.060, [0.056–0.063]) or ALBI-LP (0.060, [0.057–0.064]), and equivalent to h-HUI by ICG-PDR (0.051, [0.045, 0.055]).

**Conclusion:**

The HUI harmonized by the ALBI-LP is a clinically applicable method for ensuring the comparability of MR devices in quantitative liver reserve prediction using gadoxetate disodium-enhanced MR imaging.

**Key Points:**

***Question*** *The accurate prediction of quantitative liver function by hepatocyte-specific contrast-enhanced MRI necessitates the harmonization of MR systems. However, no established method has yet been identified.*

***Findings*** *In quantitative hepatic function assessment, albumin-bilirubin linear predictor can be employed to achieve harmonization between MR systems equivalent to the indocyanine green clearance test.*

***Clinical relevance*** *Quantitative liver function, as measured by the indocyanine green clearance test, can be accurately estimated using the hepatocellular uptake index, harmonized with the albumin-bilirubin linear predictor, across diverse MR systems.*

**Graphical Abstract:**

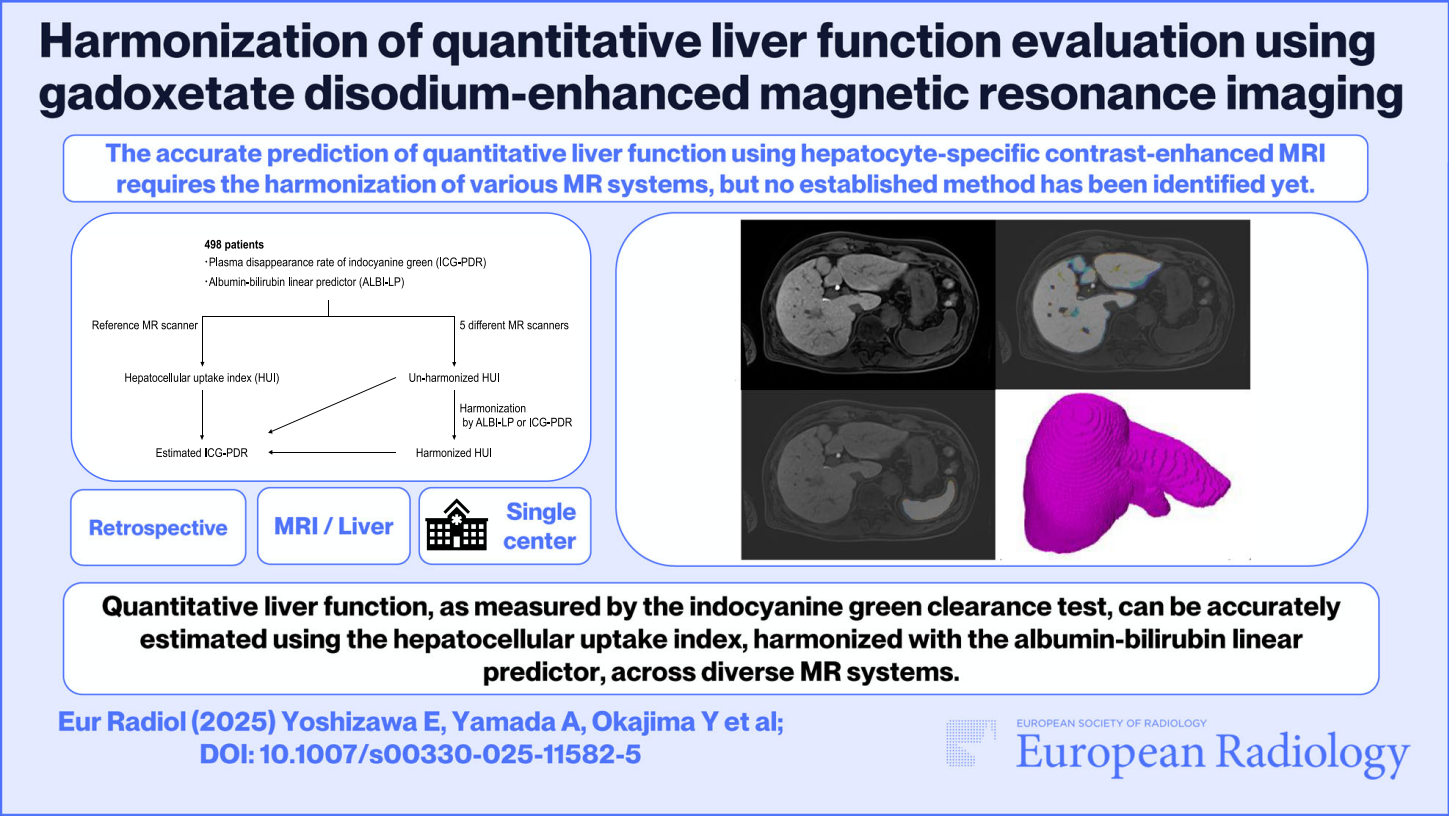

## Introduction

Liver resection remains a cornerstone treatment for hepatic neoplasms. However, the risk of post-hepatectomy liver failure (PHLF) poses a significant challenge [1]. While the indocyanine green (ICG) clearance test is a widely employed method for assessing hepatic reserve preoperatively and is routinely performed in several countries [2, 3], it exhibits limitations, including allergic reactions, anaphylactic shock, and an inability to evaluate partial hepatic function. Thus, alternative criteria for predicting safe resection cutoff levels have been proposed [4].

The albumin-bilirubin linear predictor (ALBI-LP) provides a valuable, easily available alternative for predicting hepatic reserve. ALBI scores have been demonstrated to correlate well with the ICG clearance test [5] and are frequently used worldwide [6]. However, both the plasma disappearance rate of indocyanine green (ICG-PDR) and ALBI-LP have limitations because they cannot assess partial liver function. Given that liver function is not always uniform across the liver, particularly in patients with chronic liver disease or cirrhosis, partial liver function assessments, including anatomical assessments, are essential to predict postoperative liver failure.

Gadoxetate disodium-enhanced magnetic resonance (MR) imaging (EOB-MRI) has been proposed as a means of evaluating hepatic functional reserve, given that specific uptake by hepatocytes reflects their function [7, 8]. The application of EOB-MRI for the quantitative assessment of partial liver reserve, particularly for the prediction of PHLF, represents a significant clinical challenge. Several indices have been proposed for clinical use [9–21], with the hepatocellular uptake index (HUI) being a promising quantitative imaging index of liver reserve.

However, a major limitation in the clinical dissemination of quantitative MR imaging biomarkers based on MR signal intensities is the variability of these intensities depending on the scanner and imaging parameters employed [22, 23]. To overcome this limitation, standardization methods such as relaxometry have been attempted, but they have not been widely implemented due to technical constraints.

Harmonization, a metrological approach that enhances measurement consistency when standardization is challenging, offers a potential solution [24–26]. This study aimed to develop a clinically applicable harmonization methodology for HUI, a quantitative index of liver function, using EOB-MRI. Such a methodology would enable the consistent application of HUI across diverse MR systems, facilitating its integration into clinical practice.

## Materials and methods

### Subjects

This retrospective study was approved by our institutional review board, and the requirement for informed consent was waived. This study was performed in accordance with the ethical standards as laid down in the 1964 Declaration of Helsinki and its later amendments or comparable ethical standards. Data from consecutive patients who underwent three-dimensional gradient-echo (GRE) T1-weighted EOB-MRI and HUI measurements as preoperative evaluation between April 2011 and June 2024 at our institution were extracted from the radiological report database.

Two subject groups were then generated for different harmonization strategies based on the presence or absence of the ICG clearance test and sufficient blood biochemical data to calculate ALBI-LP within 1 month of MR.

### MR imaging

The entire liver and spleen were imaged 20 min after intravenous administration of 0.025 mmol/kg or 0.1 mL/kg body weight hepatobiliary contrast material (EOB Primovist injection syringes 5 or 10 mL, Bayer) using single-breath-hold three-dimensional GRE or fat suppression to measure HUI on six different MR systems from the same manufacturer (Siemens Healthcare). Four 3-Tesla (T) scanners (Trio Tim, Magnetom Vida, and two Magnetom Prisma) and two 1.5-T scanners (Avanto and Avanto fit) were used during the study period. The parameters of each MR system are presented in the Supplementary Materials (Table S1).

### Image analysis

During the study period, two board-certified radiologists (E.Y. and A.Y.) with more than 10 years of experience in diagnostic imaging independently calculated the HUI and recorded it in their radiological reports as part of their daily practice. The HUI was calculated from the volume and mean signal intensity of the liver (V_L_, L_20_) and the mean signal intensity of the spleen (S_20_) on contrast-enhanced T1-weighted images with fat suppression, 20 min after EOB administration. HUI can be calculated using the formula HUI = V_L_ (L_20_/S_20_ − 1) [9] with custom analysis software in MATLAB (MathWorks) [18]. The liver area was measured by the semi-automatic segmentation of the transverse MR images, and the liver volume was calculated by multiplying the area of the liver by the slice thickness (Fig. S2). Although the HUIs measured in routine practice were used for the analysis in this study, 50 randomly selected cases were independently evaluated by a hepatologist with over a decade of experience in liver surgery (T.N.) to ascertain the concordance of the HUI measurement.

### Laboratory tests

A dose of 0.5 mg/kg ICG was administered intravenously, and blood sampling was performed at 5-, 10-, and 15-min intervals following ICG administration. The ICG-PDR was determined by regression analysis [9]. Simultaneously, serum total bilirubin and albumin levels, and the ALBI-LP were measured [27]. The median duration between the MR scan and laboratory test results was 0 days (interquartile range: 0–3).

### Harmonization strategy

The signal intensity of MRI is not standardized in the same way as the Hounsfield Unit in CT, and the scaling of signal intensity differs between devices from different vendors [28]. As a result, it is not possible to directly compare the indices derived from the obtained signal intensity. In situations where standardization (obtaining the same results even with different devices) is difficult, harmonization is the process of adjusting the obtained results so that they can be compared. Harmonization facilitates the sharing of results across different vendors and devices, making it a crucial element for promoting large-scale clinical research, such as multicenter collaborative studies [24]. The actual harmonization procedure in this study is as follows.

**Fig. 1:**
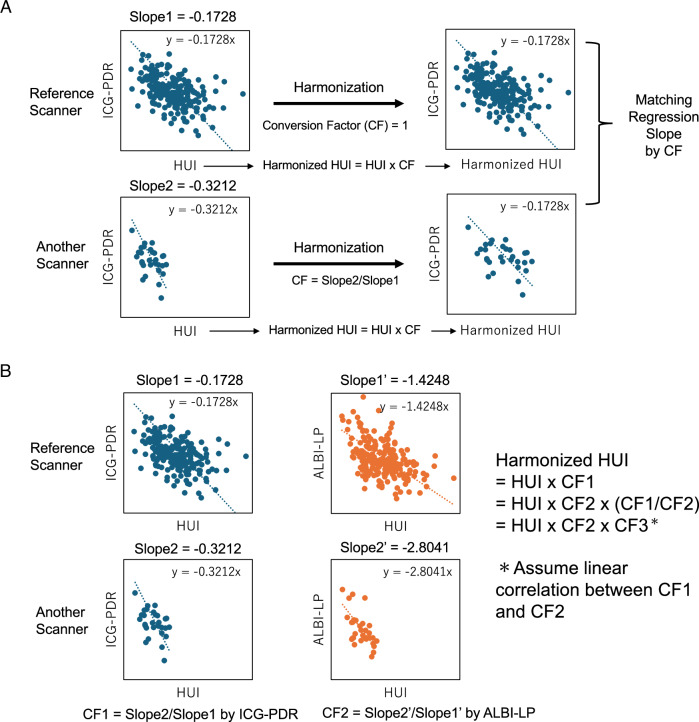
**A** Harmonization method for HUI obtained with different MR systems using ICG-PDR. HUI, hepatocellular uptake index; ICG-PDR, indocyanine green plasma disappearance rate; CF, conversion function for harmonization; MR, magnetic resonance. **B** Harmonization method for HUI obtained with different magnetic resonance systems using ALBI-LP. HUI, hepatocellular uptake index; MR, magnetic resonance; ICG-PDR, indocyanine green plasma disappearance rate; ALBI-LP, albumin-bilirubin linear predictor, CF1, conversion function using ICG-PDR; CF2, conversion function using ALBI-LP; CF3, conversion function between CF1 and CF2

The slope of the linear regression without the intercept between HUI and ICG-PDR obtained from the reference MR system was set to Slope1, and that obtained from the other MR system was set to Slope2. A conversion factor (CF1) expressed as Slope2/Slope1 was defined so that harmonized HUI (h-HUI) could be obtained by h-HUI = HUI･CF1 (Fig. 1A).

Similarly, the slope of the linear regression without the intercept between HUI and ALBI-LP obtained from the reference MR system was set to Slope1’, and that obtained from other MR systems was set to Slope2’. Then, a conversion factor (CF2) expressed as Slope2’/Slope1’ was defined. If a linear proportional relationship existed between CF1 and CF2, the coefficient CF3 (CF3 = CF1/CF2) was set, and h-HUI could be obtained using CF2 and CF3 even if the value of CF1 was unknown (h-HUI = HUI･CF1 = HUI･CF2･CF3) (Fig. 1B).

Finally, estimated ICG-PDR (eICG-PDR) could be calculated using the equation: eICG-PDR = Slope1･h-HUI.

According to this harmonization strategy, CF1 (= Slope2/Slope1), CF2 (= Slope2’/Slope1’), and CF3 (= CF1/CF2) were determined for each MR system. The MR system that performed the largest number of examinations (Trio Tim) was used as the reference MR system in this study.

### Statistical analysis

The concordance of HUI measurements between observers was evaluated by calculating intraclass correlation coefficient (ICC) between radiologists and surgeon. To verify the commutability and accuracy of the h-HUI using ALBI-LP as a quantitative index of the hepatic functional reserve, the following statistical analyses were performed. First, linear regression analysis was performed between the HUI and ICG-PDR for each MR system. The 95% confidence interval (95% CI) of the slope of the zero-intercept regression equation between h-HUI and ICG-PDR for the reference MR system and each MR system was determined. If an overlap of 95% CIs was observed between two slopes obtained from different MR systems, commutability between the two systems was considered. Second, the accuracy of predicting the quantitative hepatic functional reserve corresponding to ICG-PDR using various indices, including h-HUI by ICG-PDR or ALBI-LP, non-harmonized HUI, and ALBI-LP, was examined by calculating the root mean square error (RMSE) and analyzing the distribution analysis of residuals between the observed ICG-PDR and eICG-PDR.

All statistical analyses were performed using MATLAB (MathWorks). No overlap in 95% CIs or *p*-values less than 0.05 were considered to indicate statistical significance.

## Results

**Fig. 2:**
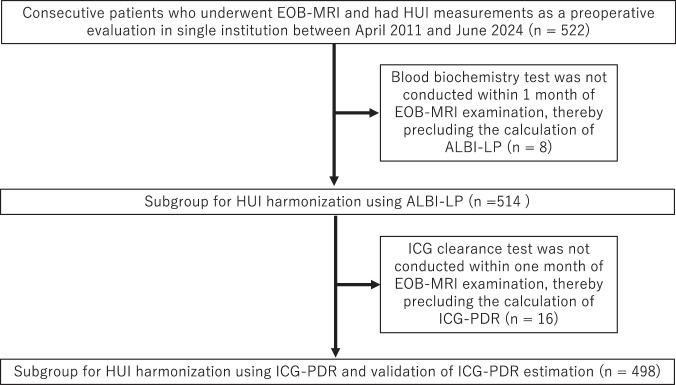
Flow diagram of included subjects in this study. EOB-MRI, gadoxetate disodium-enhanced MR; HUI, hepatocellular uptake index; ICG-PDR, indocyanine green plasma disappearance rate; ALBI-LP, albumin-bilirubin linear predictor

A total of 522 consecutive patients (336 men, 186 women; mean age, 67.3 years ± 12.5) who underwent EOB-MRI and had HUI measurements as a preoperative evaluation in our institution between April 2011 and June 2024 were initially included and extracted from the radiological report database. Two subgroups were then generated for different harmonization strategies: one based on the presence or absence of the ICG clearance test, and the other based on the availability of blood biochemical tests within 1 month of MR. For subgroup 1 (HUI harmonization using ICG-PDR), eight patients were excluded due to the absence of ICG-PDR measurements. In subgroup 2 (HUI harmonization using ALBI-LP), 16 patients were excluded due to the absence of both ICG-PDR and ALBI-LP measurements. Details of the flow diagram (Fig. 2) and demographic table (Table 1) are provided.

**Table 1:** Demographics table

	HUI harmonization using ICG-PDR	HUI harmonization using ALBI-LP
Number	498	514
Sex		
Male	320	330
Female	178	184
Age	68.0 ± 11.6	67.5 ± 12.3
Liver function		
ICG-PDR	−0.16 ± 0.04	-
ALBI-LP	−1.32 ± 0.55	−1.33 ± 0.56
Magnetic resonance scanner		
Avanto	31	31
Avanto Fit	38	40
Vida	23	24
Prisma1	174	180
Prisma2	36	38
Trio Tim	196	201

*HUI* hepatocellular uptake index, *ICG-PDR* indocyanine green plasma disappearance rate, *ALBI-LP* albumin-bilirubin linear predictor

**Fig. 3:**
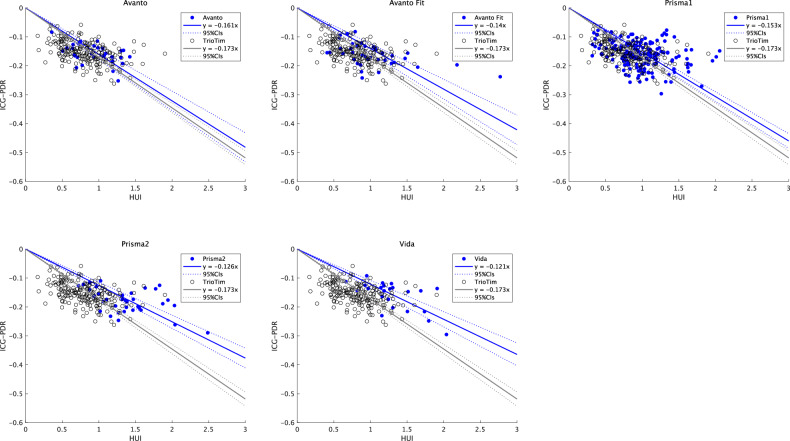
Linear regression analysis of ICG-PDR and non-harmonized HUI in various MR systems. Significant differences in regression slope were identified between the reference magnetic resonance system (Trio Tim) and most of the other magnetic resonance systems (4/5). ICG-PDR, indocyanine green plasma disappearance rate; HUI, hepatocellular uptake index; MR, magnetic resonance

The ICC (2,1) between observers was 0.97. A statistically significant linear correlation was observed between the HUI specific to each MR system and the ICG-PDR (Table 2, Fig. 3). However, no overlap was observed between the 95% CI of the slope calculated from most other MR systems (4/5) and the 95% CI of the slope of the reference MR system.

**Table 2:** The slope of linear regression of ICG-PDR by HUI and CF1

MR scanner	Slope (95% CIs)	*p*-value	CF1
Avanto	−0.161 (−0.177, −0.144)	< 0.001	0.930
Avanto Fit	−0.141 (−0.158, −0.124)*	< 0.001	0.813
Vida	−0.121 (−0.135, −0.108)*	< 0.001	0.702
Prisma1	−0.153 (−0.162, −0.145)*	< 0.001	0.887
Prisma2	−0.126 (−0.137, −0.114)*	< 0.001	0.727
Trio Tim	−0.173 (−0.181, −0.165)	< 0.001	1 (reference)

*ICG-PDR* indocyanine green plasma disappearance rate, *HUI* hepatocellular uptake index, *CF1* conversion function for harmonization by ICG-PDR

* Significantly different from the reference MR scanner (Trio Tim)

**Fig. 4:**
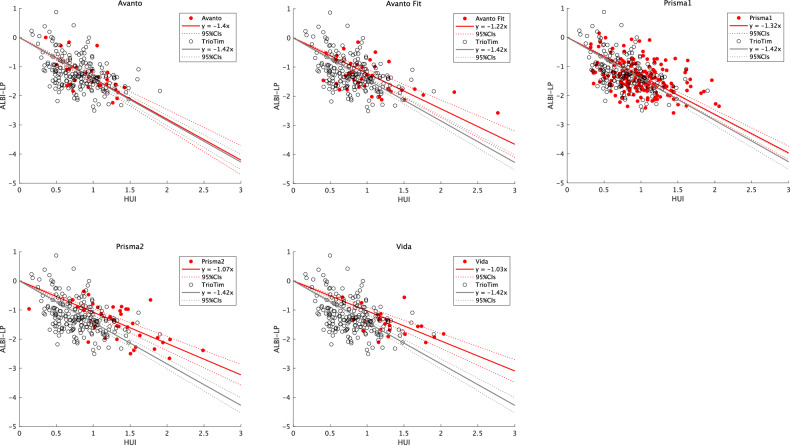
Linear regression analysis of ALBI-LP and non-harmonized HUI in various MR systems. A similar tendency of differences in regression slope was identified between the reference magnetic resonance system (Trio Tim) and other magnetic resonance systems. HUI, hepatocellular uptake index; MR, magnetic resonance; ALBI-LP, albumin-bilirubin linear predictor

Similarly, a statistically significant linear correlation and similar tendency in the regression slope were observed between the HUI specific to each MR system and ALBI-LP (Table 3, Fig. 4).

**Table 3:** The slope of linear regression of ALBI-LP by HUI and CF2

MR scanner	Slope (95% CIs)	*p*-value	CF2
Avanto	−1.402 (−1.569, −1.235)	< 0.001	0.984
Avanto Fit	−1.216 (−1.367, −1.066)	< 0.001	0.854
Vida	−1.031 (−1.160, −0.902)*	< 0.001	0.723
Prisma1	−1.324 (−1.401, −1.246)	< 0.001	0.929
Prisma2	−1.075 (−1.194, −0.956)*	< 0.001	0.754
Trio Tim	−1.425 (−1.511, −1.338)	< 0.001	1 (reference)

*ALBI-LP* albumin-bilirubin linear predictor, *HUI* hepatocellular uptake index, *CF2* conversion function for harmonization by ALBI-LP

* Significantly different from the reference MR scanner (Trio Tim)

From the five MR systems except for the reference MR system, a statistically significant linear correlation was observed between CF1 and CF2 (CF1 = 0.955･CF2, *p* < 0.001), and CF3 was determined to be CF1/CF2 = 0.955 (Fig. S1).

According to the harmonization strategy described above, the following equations calculating h-HUI and eICG-PDR were determined: h-HUI = HUI･(Slope2/−0.173), h-HUI = HUI･(Slope2’/−1.425)･0.955, and eICG-PDR = −0.173･h-HUI, where Slope2 and Slope2’ are the linear regression slopes for HUI by ICG-PDR and ALBI-LP at individual institutions, respectively.

**Fig. 5:**
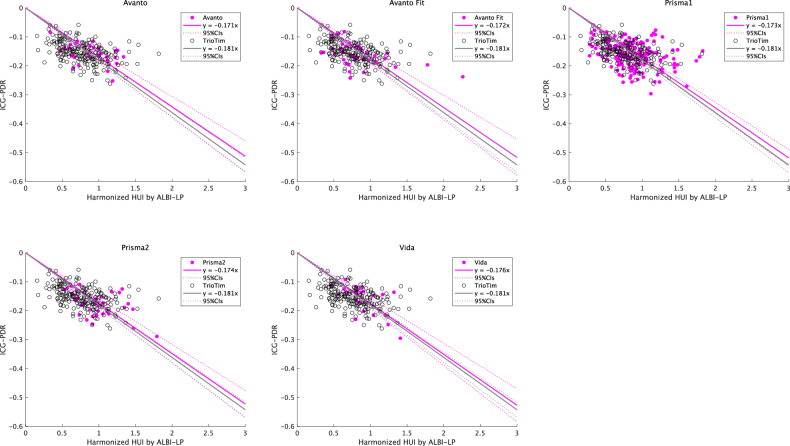
Linear regression analysis of ICG-PDR and Harmonized HUI using ALBI-LP on various MR systems. No statistically significant differences in regression slopes were identified between the reference magnetic resonance device (Trio Tim) and the other magnetic resonance devices as a result of harmonization. HUI, hepatocellular uptake index; MR, magnetic resonance; ALBI-LP, albumin-bilirubin linear predictor; ICG-PDR, indocyanine green plasma disappearance rate

The 95% CI of the slope in the regression equation between the ICG-PDR and h-HUI harmonized by ALBI-LP overlapped with that of the reference MR system for all five MR scanners (Table S2, Fig. 5).

The RMSE for estimating ICG-PDR was not significantly different between the h-HUIs harmonized by ICG-PDR and ALBI-LP. Conversely, statistically significant larger errors compared to the reference MR system (Trio Tim) were identified in the non-harmonized HUI obtained from one MR system (Vida) and ALBI-LP.

According to the distribution analysis of residuals in ICG-PDR estimation using normal distribution fitting, h-HUI by ICG-PDR exhibited small μ and σ, indicating a small bias and good precision. Here, μ and σ represent the mean and standard deviation of the approximated normal distribution of the residuals.

Individual HUI before harmonization (Vida) demonstrated a large μ but a small σ, suggesting a large bias but good precision. Overall, the HUI before harmonization displayed both large μ and σ, indicating a large bias and poor precision.

ALBI-LP, on the other hand, exhibited a small μ but a large σ, suggesting a small bias but poor precision.

**Fig. 6:**
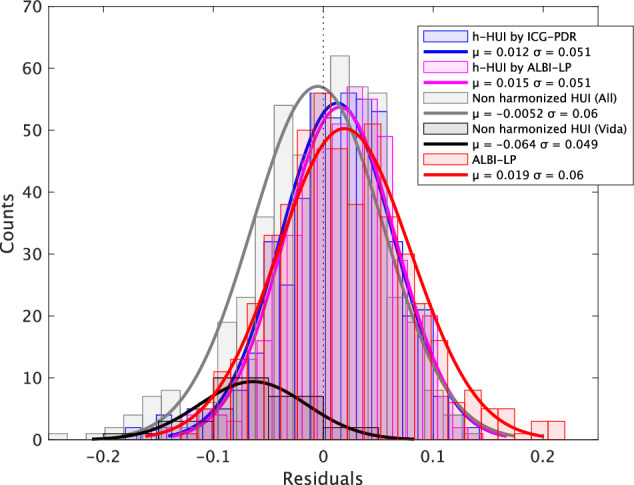
Distribution analysis of residuals in ICG-PDR estimation using various methods. The residuals between observed and estimated ICG-PDR were approximated by a normal distribution. HUI harmonized by ICG had small μ and σ (small bias, good precision). Individual HUI before harmonization (Vida) had large μ but small σ (large bias but good precision). Overall HUI before harmonization had large μ and σ (large bias and poor precision). ALBI-LP had small μ but large σ (small bias but poor precision). HUI after harmonization with ALBI-LP had small μ and σ (small bias, good precision). Where μ and σ are the mean and standard deviation of the approximated normal distribution of the residuals. HUI, hepatocellular uptake index; ALBI-LP, albumin-bilirubin linear predictor; ICG-PDR, indocyanine green plasma disappearance rate

Finally, HUI after harmonization with ALBI-LP demonstrated both small μ and σ, indicating a small bias and good precision (Table 4, Fig. 6).

**Table 4:** Distribution analysis of residuals in estimating ICG-PDR

Methods	RMSE (95% CIs)	μ (95% CIs)	σ (95% CIs)
h-HUI by ICG-PDR	0.042 (0.039, 0.045)	0.012 (0.008, 0.017)	0.051 (0.048, 0.055)
h-HUI by ALBI-LP	0.042 (0.040, 0.045)	0.015 (0.011, 0.019)	0.051 (0.048, 0.054)
Non-harmonized HUI (All)	0.046 (0.043, 0.050)	−0.005 (−0.010, 0.000)*	0.060 (0.056, 0.063)*
Avanto	0.039 (0.030, 0.048)	−0.002 (−0.019, 0.015)	0.046 (0.037, 0.061)
Avanto Fit	0.048 (0.033, 0.064)	−0.014 (−0.036, 0.008)	0.067 (0.054, 0.086)
Vida	0.067 (0.046, 0.086)*	−0.064 (−0.085, −0.043)*	0.049 (0.038, 0.069)
Prisma1	0.049 (0.043, 0.055)	−0.006 (−0.015, 0.003)*	0.062 (0.056, 0.069)*
Prisma2	0.061 (0.043, 0.080)	−0.056 (−0.076, −0.035)*	0.061 (0.049, 0.079)
Trio Tim (reference)	0.040 (0.036, 0.044)	0.013 (0.006, 0.020)	0.049 (0.044, 0.054)
ALBI-LP	0.049 (0.046, 0.053)*	0.019 (0.014, 0.024)	0.060 (0.057, 0.064)*

μ and σ are the mean and standard deviation of the approximated normal distribution of the residuals

*ICG-PDR* indocyanine green plasma disappearance rate, *ALBI-LP* albumin-bilirubin linear predictor, *HUI* hepatocellular uptake index, *h-HUI* harmonized HUI, *RMSE* root mean square error

* Significantly different from the reference MR scanner (Trio Tim)

## Discussion

The major findings of this study are as follows. (1) A significant linear correlation was observed in every MR system between the ICG-PDR and the HUI; however, poor commutability was observed between MR systems. (2) An h-HUI equivalent to that obtained using ICG-PDR can be calculated using ALBI-LP with the following equation: h-HUI = HUI･(Slope2’/−1.425)･0.955, where Slope2’ is the linear regression slope between HUI and ALBI-LP at the individual MR system. (3) The quantitative liver function corresponding to ICG-PDR can be accurately estimated using the equation eICG-PDR = −0.173･h-HUI, which is superior to non-harmonized HUI or ALBI-LP itself.

It is noteworthy that the regression line between the reference standard for liver function (ICG-PDR) and the liver function index (HUI) obtained from MRI exhibited a slope that was significantly different from the reference standard MR device, even when the devices were the same model (Prisma1 and Prisma2). However, a non-significant difference was observed between the HUI harmonized using ALBI-LP and the ICG-PDR. This finding indicates that variations in signal intensity can occur when different MR devices are used, even when they are the same model. It has been documented that discrepancies in magnetic field non-uniformity and geometric distortion can arise due to manufacturing processes and the effects of aging deterioration, even for models of the same design [25, 29]. Therefore, it is essential to harmonize HUI to disseminate it as a quantitative MR biomarker that can be used worldwide for PHLF prevention. According to our results, in institutions where the ICG clearance test was performed, accurate harmonization can be achieved using CF1 (= Slope2/Slope1), which can be calculated from the known Slope1 (−0.173) and the regression slope between ICG-PDR and HUI obtained from EOB-MRI performed on the institution’s MR system (Slope2).

Our finding of a significant linear correlation between CF1 and CF2 obtained from different MR systems is key to developing a clinically applicable harmonization methodology without an ICG clearance test. The conversion factor CF3 (= CF1/CF2) could be set using the ratio of CF1 and CF2, allowing CF1 to be replaced by CF2･CF3 and HUI to be harmonized solely using ALBI-LP. This correlation may have been obtained because the ICG-PDR, ALBI-LP, and HUI are indices designed to assess liver function as a linear function with an intercept of 0.

From a metrological perspective, the accuracy of quantitative imaging biomarkers should be discussed in terms of their trueness (nonbiased) and precision. Notably, our method achieved accurate (low bias and good precision) harmonization of easily biased MR signal intensity-based indices, such as HUI, using poorly precise but low-cost clinical parameters, such as ALBI-LP, without using accurate but high-cost hepatic function tests, such as the ICG clearance test. Our distribution analysis of residuals in estimating ICG-PDR revealed that HUI is easily biased by the difference of MR systems; however, individual HUI had good precision (small σ). On the other hand, ALBI-LP had poor precision (large σ) but a small bias (small μ). In other words, efficient harmonization can be achieved by combining an index with a large bias but good precision, such as HUI, and an index with poor precision but a small bias, such as ALBI-LP.

This harmonization strategy is expected to be applied to other quantitative MR biomarkers, in addition to HUI, especially for the estimation of partial liver function. Because the HUI targeted by the harmonization method proposed in this study is an indicator of total liver function corresponding to ICG-PDR, the effect of the proposed method itself on the evaluation of partial liver function was not directly verified. However, the proposed method may indirectly improve the accuracy of partial liver function assessment because the conversion factor determined in this study can be applied to the indicator of partial liver function, rHUI (remnant hepatocellular uptake index) [9, 14, 16, 18], measured by different MR scanners. Finally, we confirmed that h-HUI with ALBI-LP predicted liver function corresponding to ICG-PDR more accurately than non-harmonized HUI or ALBI-LP itself. In other words, h-HUI using the ALBI-LP can predict the PHLF more accurately than non-harmonized HUI. Thus, our harmonizing methodology could be a powerful tool for future multicenter clinical studies. In addition to metrological standardization and harmonization, attempts have been made to standardize the HUI according to individual patient factors such as body surface area [17, 30]. By combining the scanner-dependent harmonization method proposed in this study with the patient-dependent standardization method, it may be possible to more accurately predict residual liver function.

Our study has several limitations, including a small and non-uniform study population and a retrospective study design. However, from a clinical application perspective, it is noteworthy that our harmonization methodology was effective, even with a non-uniform study population.

Future studies should determine the optimal number of patients with a certain level of hepatic functional reserve required for more accurate harmonization.

The h-HUI by the ALBI-LP represents a clinically applicable methodology to ensure the commutability of MR devices for quantitative liver reserve prediction using gadoxetate disodium-enhanced MR imaging.

## Supplementary information

Supplementary information

Supplementary information

Supplementary information

## Abbreviations

ALBI-LP: Albumin-bilirubin linear predictor
CF1: Conversion factor for HUI harmonization using ICG-PDR
CF2: Conversion factor for HUI harmonization using ALBI-LP
CF3: Conversion factor between CF1 and CF2 (= CF1/CF2)
eICG-PDR: Estimated ICG-PDR
EOB-MRI: Gadoxetate disodium-enhanced MR imaging
GRE: Gradient-echo
h-HUI: Harmonized HUI
HUI: Hepatocellular uptake index
ICG: Indocyanine green
ICG-PDR: Plasma disappearance rate of indocyanine green
MR: Magnetic resonance
PHLF: Post-hepatectomy liver failure
RMSE: Root mean square error
