# Supplementary figures and images for: Harmonization of quantitative liver function evaluation using gadoxetate disodium-enhanced magnetic resonance imaging
Source: Eur Radiol. 2025 Apr 18;35(11):7372–81. doi: 10.1007/s00330-025-11582-5 (PMC12559099; doi:10.1007/s00330-025-11582-5)

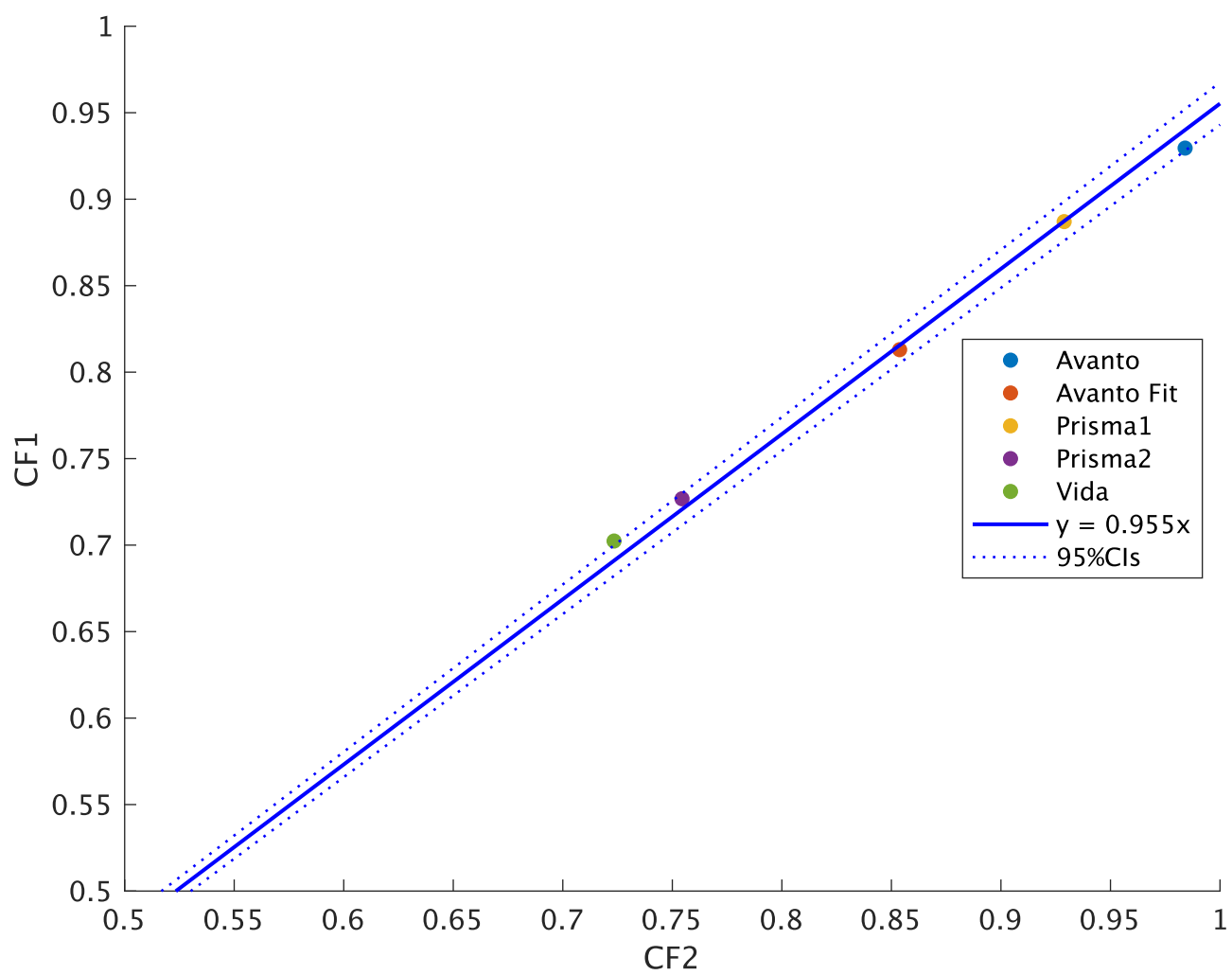

Supplement: Supplementary file 1 — Supplementary information [file 330_2025_11582_MOESM1_ESM.pdf]

a

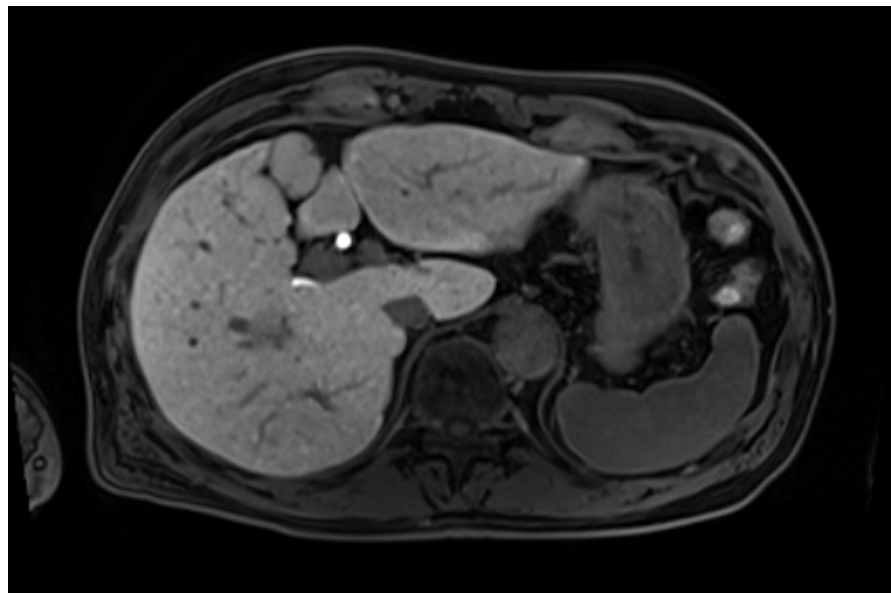

b

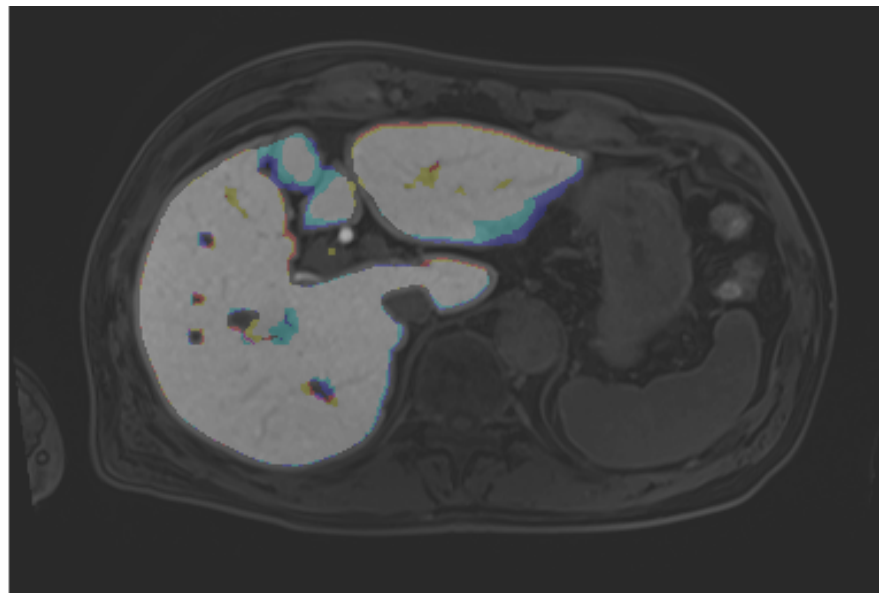

c

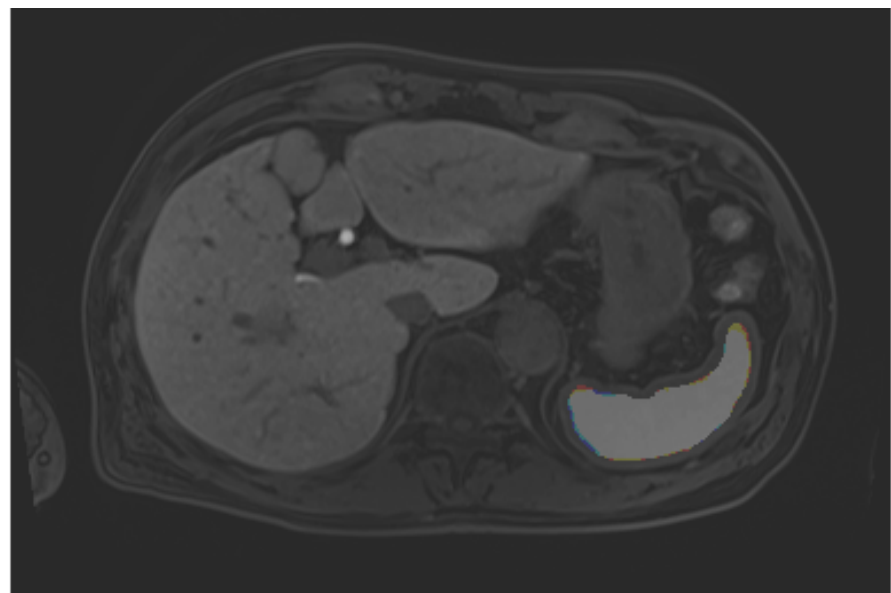

d

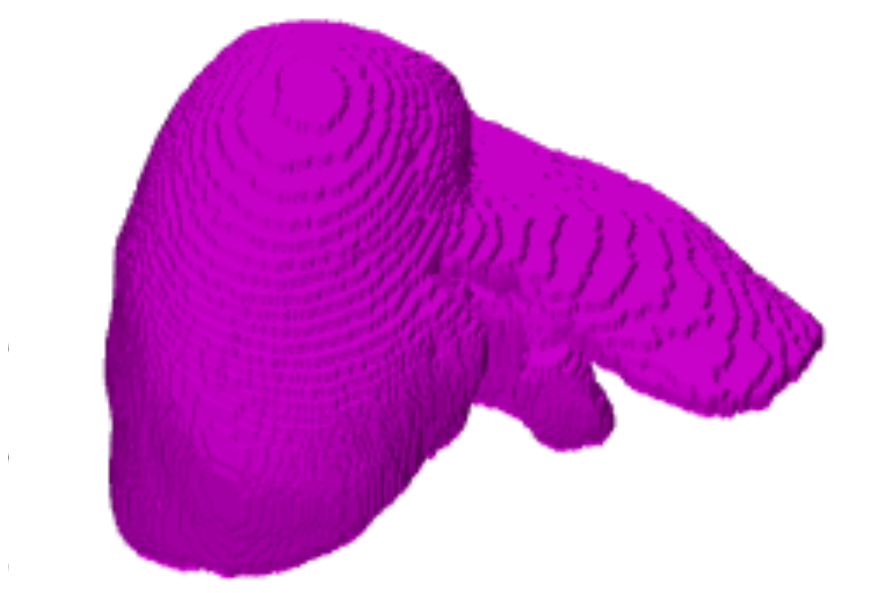

Supplement: Supplementary file 2 — Supplementary information [file 330_2025_11582_MOESM2_ESM.pdf]
