# Supplementary material for: Harmonization of quantitative liver function evaluation using gadoxetate disodium-enhanced magnetic resonance imaging
Source: Eur Radiol. 2025 Apr 18;35(11):7372–81. doi: 10.1007/s00330-025-11582-5 (PMC12559099; doi:10.1007/s00330-025-11582-5)
Supplement: Supplementary file 3 — Supplementary information [file 330_2025_11582_MOESM3_ESM.docx]

**Table S1.** The parameters of each MR systems

| Scanner | Magnetic Field Strength [T] | Repetition Time [ms] | Echo Time [ms] | Flip Angle [degree] | Slice Thickness [mm] | Field of View [mm] | Acceleration Factor |
| --- | --- | --- | --- | --- | --- | --- | --- |
| TrioTim | 3.0 | 3.0 | 1.23 | 14 | 3.0 | 322x429 | 2 |
| Prisma1* | 3.0 | 3.36 | 1.17 | 13 | 2.5 | 296x379 | 4 |
| Prisma2* | 3.0 | 3.36 | 1.17 | 13 | 2.5 | 296x379 | 4 |
| Vida | 3.0 | 3.37 | 1.19 | 13 | 2.5 | 296x379 | 4 |
| Avanto | 1.5 | 4.05 | 1.86 | 15 | 3.0 | 275x400 | 2 |
| Avanto-fit | 1.5 | 3.3 | 1.2 | 13 | 2.5 | 296x379 | 4 |

* Two of the same models.

**Table S2.** The slope of linear regression of ICG-PDR by harmonized HUI using ALBI-LP

| **MR scanner** | **Slope (95% CIs)** |
| --- | --- |
| Avanto | -0.171 (-0.189, -0.153) |
| Avanto Fit | -0.172 (-0.193, -0.151) |
| Vida | -0.176 (-0.189, -0.157) |
| Prisma1 | -0.173 (-0.182, -0.163) |
| Prisma2 | -0.174 (-0.190, -0.159) |
| Trio Tim* | -0.173 (-0.181, -0.165) |

ICG-PDR, indocyanine green plasma disappearance rate; ALBI-LP, albumin-bilirubin linear predictor; HUI, hepatocellular uptake index. *Reference magnetic resonance scanner.
